# Impact Factors on Oven-Dry Density Measurements of Wood

**DOI:** 10.3390/ma18184396

**Published:** 2025-09-20

**Authors:** Lukas Emmerich, Moritz Kampherm, Christian Brischke

**Affiliations:** 1 Wald und Holz NRW—Centre of Forest and Wood-Based Industries (FBV), Team Wood-Based Industries, Carlsauestraße 91A, D-59939 Olsberg, Germany; 2 Wood Biology and Wood Products, Faculty of Forest Sciences and Forest Ecology, University of Goettingen, Büsgenweg 4, D-37077 Goettingen, Germany; moritz.kampherm@stud.uni-goettingen.de; 3 Thünen Institute of Wood Research, Leuschnerstraße 91d, D-21031 Hamburg, Germany; christian.brischke@thuenen.de

**Keywords:** absolute dry density, durability testing, evaporation, hardwoods, softwoods

## Abstract

Wood density is a key property since it affects almost every other property of wood such as its elasto-mechanical, acoustic, thermal, or electrical properties. Hence, it is essential to determine wood density for the interpretation of any other property test. Density measurements are usually carried out gravimetrically by measuring the wood specimens’ dimensions and taking their weight. In order to be independent of moisture, wood density is measured at an absolute dry state. However, depending on which wood properties shall be measured after the oven-dry density is determined, heating the wood up to 103 °C can be problematic because the volatile components of the wood can evaporate. For this reason, the drying conditions (temperature in °C (60, 80, 103 °C)), duration in h (8, 16, 24, 48 h)) required to achieve an absolute dry state inside wood specimens—being obligatory for the analysis of various physical, mechanical, or even biological properties—were examined for different softwood and hardwood species. Basically, oven-dry measurements (i.e., 48 h at 103 °C) themselves contained a significant error, which was considered to be the result of deviations in the handling of the specimens and the scales used. Using temperatures below 103 °C was critical for the determination of absolute dry mass and dimensions. Wood specimens with a high content of volatile ingredients led to an apparently increased residual MC (e.g., shown for Scots pine heartwood), thus volatile ingredients were considered an additional source of error during oven-dry measurements.

## 1. Introduction

Density (ρ) describes the relationship between the mass and volume of a material and influences numerous other material properties such as elasto-mechanical, acoustic, thermal, or electrical properties (e.g., [1,2,3,4]). In wood materials science, it is therefore essential to determine the density of wood whenever other material parameters are tested in order to be able to adequately interpret the latter. Density itself is affected by changes in material moisture content, as can be observed with porous and/or hygroscopic materials. Thus, water states and dynamics severely affect the calculation of density and consequently the use of wood-based products and their material properties, i.e., mechanical characteristics (i.e., [5,6,7,8,9]) or degradation processes by decaying fungi alter with changing moisture content (i.e., [10]). In particular, water can exist in (1) liquid, (2) gaseous and (3) sorbed states in a hygroscopic material such as wood, whereas moisture transport occurs throughout multiple pathways within cell walls (cell wall water) and in the macro void structure (capillary water). In particular, sorbed water describes the state at which water molecules are energetically bound to the wood polymers (especially hemicelluloses and celluloses) via free hydroxyl groups (‘sorption sides’), i.e., by hydrogen bonds or other intermolecular mechanisms [11]. In principle, the uptake of water molecules in the bulk of the cell walls and the capillary structure is referred to as a ‘sorption process’. Thereby, ‘absorption’ describes the process by which water molecules move from the vapor phase to water that is associated with the wood polymers (sorbed water) and ‘desorption’ as the vice versa process.

Being a natural, porous, hygroscopic, and anisotropic material, wood essentially consists of the cell walls and the cavities they surround, the cell lumens. Basically, the cell walls of wood are virtually non-porous at an oven-dry state with a moisture content (MC) = 0% [12]. Under changing surrounding environments (relative humidity, temperature), cell walls are able to absorb water molecules within the material structure from ambient air, while the amount absorbed is quantified by the moisture content (mass of water in relation to the oven-dry mass; [13]). According to [11], such absorption processes of water molecules within the cell walls result in an increase in the dimensions and volume of the wood. Conversely, the wood shrinks as water is released from the wood cell walls by the process called desorption. However, the processes of absorption and desorption are limited to the hygroscopic range, i.e., at the relative humidity (RH) of the ambient air between 0%, the absolute dry state, and 100%, the water-saturated state. In equilibrium with the latter, the wooden cell walls become moisture-saturated. In theory, cell wall saturation (CWS, formerly: fiber saturation) is achieved when all cell walls are filled with the maximum amount of water molecules, but the lumens are still free of water. Principally, CWS describes the threshold moisture content above which physical properties do not experience significant changes with increasing moisture content [13]. More likely, absorption and desorption are temporarily and spatially ongoing processes which never attain to an equilibrium [9] and cell walls are not saturated with water until the whole wood specimen is fully water-saturated, which contradicts the long-held dogma that cell walls are saturated before significant amounts of capillary water are present in wood, as shown in experiments by Fredriksson and Thybring [13,14].

In addition to the water that is bound in the cell walls (‘sorbed water’), wood can take up moisture in gaseous and liquid form in the cell lumens. Principally, moisture transport in wood is a complex process since the transport of water vapor by diffusion alternates with the capillary transport of liquid water [15,16,17,18]. In addition, wood is an anisotropic material, so its properties differ between the anatomical directions of the wood. This anisotropy is primarily due to the different orientation of different cell elements in the wood tissue, different cell wall thicknesses, and different fibril angles of the cellulose in the anatomical directions [19,20,21,22].

Traditionally [23], sorption as well as density measurements have most commonly been carried out gravimetrically by measuring the wood specimens’ dimensions and taking their weight. Thereby, moisture content levels differ along cross sections of a tree (i.e., [24,25,26]), between juvenile and mature wood (MC_heartwood_ ≤ MC_sapwood_), and depending on the harvesting season [27], which finally may affect density measurements (i.e., [25]). In order to be independent of the moisture, wood density is measured at an absolute dry state, i.e., an oven-dry state with a wood moisture content (MC) of 0%. According to different standards and the literature (e.g., [28,29]), the wood specimens should be dried at 103 °C or even higher at 105 ± 3 °C for ‘wood in either form’ [30] until a constant weight is reached, cooled in a desiccator, and weighed with a defined accuracy depending on the wood specimens’ size and mass.

According to ISO 13061-2 [31], one shall ‘Dry the test pieces gradually to constant mass […]’, but the standard does not provide neither guidance on the drying temperature nor the drying schedule to achieve an absolute dry mass and density. However, depending on which wood properties are to be measured after the oven-dry density is determined, heating the wood to up to 103 °C or even higher can be problematic because the volatile components of the wood can evaporate or even the ultra-structure and cellular structure can be affected as a consequence related material characteristics [32,33]. For instance, the biological durability of wood is primarily based on the inhibitory effect of wood extractives on decay organisms [34,35]. If such substances evaporate, it can be assumed that the durability will be impaired [36,37]. Therefore, the maximum drying temperature for such test timbers in wood durability testing is 60 °C, as prescribed for instance by the European standard EN 350 [38]. In addition, the loss of volatile substances distorts the results of density measurements [39]. Alternatively, the theoretical oven-dry mass can be determined by conditioning test specimens and additional moisture control specimens at a defined climate until a constant mass is achieved. Afterwards, only the moisture control specimens are oven-dried to determine their MC. The latter is used to calculate the theoretical oven-dry weight of test specimens. Due to the variability in equilibrium moisture content (EMC), the method is subject to a small amount of systematic error [40]. To avoid both systematic errors and the loss of volatile ingredients, drying temperatures below 103 °C have been suggested occasionally (e.g., [36,41,42]). Studies by Altgen et al. [43] showed that achieving the water-free state of wood and thus its actual drying density correctly under these conditions is questionable. Nonetheless drying techniques other than hot air drying at elevated temperature exist (i.e., natural air drying, vacuum drying, and microwave vacuum drying) and may retain the VOC content in solid wood specimens to higher extents and further minimize the risk of alterations in the cellular and chemical structure (i.e., [44]). Hot air drying is often the test method of choice for oven-dry density measurements on lab-scale specimens, since it serves a less elaborate method, which in addition is applicable in simple drying cabinets with air circulation, using the basic equipment available in most material testing laboratories [45].

Therefore, in this study we aimed to examine which drying conditions (temperature in °C, duration in h) are required to achieve the oven-dry state inside the test specimens of choice. The latter was studied depending on the wood species and specimen format representing different volumes and ratios between the end grain and side grain surfaces and the volumes of the specimens.

## 2. Materials and Methods

### 2.1. Wood Specimens

**Figure 1:**
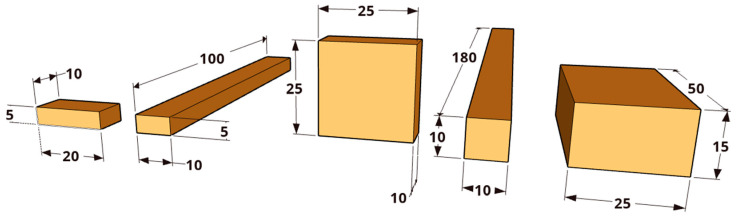
Test specimens (dimensions in mm) subjected to different drying procedures (temperature, duration), illustrated from left to right in increasing order of the specimen’s volume. Light brownish areas mark end grain surfaces.

Different test specimens were produced from European softwood and hardwood species, in particular Norway spruce (*Picea abies*), Scots pine (*Pinus sylvestris*), European beech (*Fagus sylvatica*), and black locust (*Robinia pseudoacacia*). Scots pine specimens were prepared from both sapwood and heartwood, whereas only the pure heartwood of black locust was tested. Without exception, non-kiln-dried timber was used to prepare test specimens in five different formats with different proportions of end grain surfaces and surface–volume ratios as shown in Figure 1. The selection of the specimen format was based on the requirements set by standardized and non-standardized test methods, which are applied to study the physical and elasto-mechanical properties of solid wood. Specimens of 5 × 20 × 10 (ax.) mm^3^ are commonly used to study the structural integrity of a material by High-Energy Multiple Impact (HEMI) tests [46], while specimens of 10 × 10 × 180 (ax.) mm^3^ are suggested as a standardized specimen format for 3-point-bending tests [47]. Furthermore, specimen formats used for laboratory decay tests with basidiomycete monocultures (15 × 25 × 50 (ax.) mm^3^; [48]) and those used for soft rot testing in terrestrial microcosms (5 × 10 × 100 (ax.) mm^3^; [49]) were subjects of the present study. Specimens with square-shaped end grain surfaces (e.g., 25 × 25 × 10 (ax.) mm^3^) do not illustrate a standardized specimen format but have been frequently used for testing the dimensional stability of wood in previous studies (e.g., [50]).

### 2.2. Drying Procedures

In total, 60 specimens were prepared per wood species and specimen format. Finally, these 60 specimens were divided into 12 collectives and dried under defined conditions (temperature in °C, duration in h) in drying cabinets with air circulation. Accordingly, five specimens of each species and format were exposed to 60, 80, or 103 °C for 8, 16, 24, or 48 h (Table 1), and afterwards their weight and dimensions were measured to the nearest 10^−3^ g or mm, respectively.

### 2.3. Density Measurements

Drying under controlled conditions in ventilated lab-scale drying cabinets aimed to remove water from inside the cell wall and thus achieve the oven-dry state (moisture content (*MC*) = 0%) in lab-scale wood specimens.

Non-kiln-dried wood specimens were dried according to the procedures described in Section 2.2, stored above silica gel in sealed desiccators (controlled cooling), and afterwards weighed and measured to the nearest 10^−3^ g or mm. The initial wood *MC* prior to drying was calculated according to Equation (1).

(1) $${\mathrm{MC}}_{\mathrm{initial}}\;=\frac{m_{\mathrm{initial}}\;-\;m_{\mathrm{dry}}}{m_{\mathrm{dry}}}\;\times \;100\;[\%]$$

where *MC_initial_* is the moisture content of the specimens prior to drying [%]; *m_initial_* is the mass prior to drying in ventilated drying cabinets [g]; *m_dry_* is the mass after drying in ventilated drying cabinets at defined conditions [g].

Based on a pre-test series, one confirmed that the oven-dry state in the specimen formats and wood species of the chosen sets is certain after drying at 103 °C for >36 h. The latter was approved by drying wood specimens until a constant mass was reached at 103 °C, where a constant oven-dry mass was achieved. For this reason, all specimens dried under various conditions (see Section 2.2) were subsequently exposed to a control and reference drying condition in a ventilated drying cabinet at 103 °C for 48 h and afterwards weighed and measured to the nearest 10^−3^ g or mm. On the basis of these parameters, the residual *MC* after the drying procedures described under Section 2.2 was calculated depending on the wood species and specimen format (Equation (2)).

(2) $${\mathrm{MC}}_{\mathrm{res}}\;=\frac{m_{\mathrm{dry}}-m_{0}}{m_{0}}\;\times \;100\;[\%]$$

where *MC_res_* is the moisture content of the specimens at defined conditions [%]; *m_dry_* is the mass after drying in ventilated drying cabinets at defined conditions [g]; *m*_0_ is the oven-dry mass after the control drying condition at 103 °C for 48 h [g].

The recordings of mass and dimensions of the specimens were used for the calculation of density (Equation (3)).

(3) $$\rho_{\mathrm{dry}}\;=\frac{m_{\mathrm{dry}}}{\left({\mathrm{long}}_{\mathrm{dry}}\;\times \;{\mathrm{rad}}_{\mathrm{dry}}\;\times \;{\mathrm{tang}}_{\mathrm{dry}}\right)}\;\times \;100\;[g\;{\mathrm{cm}}^{-3}]$$

where *ρ_dry_* is the density of the specimens after drying at defined conditions [g cm^−3^]; *m_dry_* is the mass after drying in ventilated drying cabinets at defined conditions [g]; *long_dry_* is the dimension in the longitudinal direction after drying at defined conditions [mm]; *rad_dry_* is the dimension in the radial direction after drying at defined conditions [mm]; *tang_dry_* is the dimension in the tangential direction after drying at defined conditions [mm].

In accordance with *MC* calculations, the actual oven-dry density was calculated based on mass and dimensions, which were measured after a controlled drying technique at 103 °C for 48 h (Equation (4)).

(4) $$\rho_{0}\;=\frac{m_{0}}{\left({\mathrm{long}}_{0}\;\times \;{\mathrm{rad}}_{0}\;\times \;{\mathrm{tang}}_{0}\right)}\;\times \;100\;[g\;{\mathrm{cm}}^{-3}]$$

where *ρ*_0_ is the actual oven-dry density of the specimens after drying at 103 °C for 48 h [g cm^−3^]; *m*_0_ is the oven-dry mass after drying at 103 °C for 48 h [g]; *long*_0_ is the dimension in the longitudinal direction after drying at 103 °C for 48 h [mm]; *rad*_0_ is the dimension in the radial direction after drying at 103 °C for 48 h [mm]; *tang*_0_ is the dimension in the tangential direction after drying at 103 °C for 48 h [mm].

Finally, the present study aimed to define the drying conditions at which the oven-dry state sets in for different specimen formats and wood species. The oven-dry density often serves as a characteristic parameter during experiments in the context of materials science. Hence, *ΔDensity* (in %) was calculated (Equation (5)), providing the percentage difference in density measurements after different drying procedures (see Section 2.2) from the actual oven-dry density of a corresponding specimen collective. Accordingly, a value of >0% would indicate residual moisture inside a wooden specimen, thus demonstrating that the applied drying conditions were not sufficient to remove moisture entirely.

(5) $$\Delta \mathrm{Density}\;=\frac{\left(\rho_{\mathrm{dry}}-\rho_{0}\right)}{\rho_{0}}\;\times \;100\;[\%]$$

where *ΔDensity* is the difference between dry density and actual oven-dry density [%]; *ρ_dry_* is the density of the specimens after drying at defined conditions [g cm^−3^]; *ρ*_0_ is the actual oven-dry density of the specimens after drying at 103 °C for 48 h [g cm^−3^].

Furthermore, the impact factors on the drying processes and oven-dry density measurements were analyzed. In this context, *ΔDensity* (%) was studied depending on the specimen’s volume, the total surface–volume ratio, the end grain area–volume ratio, and the end grain–total surface ratio and summarized in graphical illustrations.

## 3. Results and Discussion

### 3.1. Moisture Content Measurements

The residual moisture content *MC_res_* after drying under defined conditions is illustrated in Table 1 for the wood species, specimen formats, and combinations of drying temperature and duration of choice. As indicated by color gradients from dark yellow to dark green, *MC_res_* decreased generally with increasing drying temperature and duration. At a drying temperature of 60 °C, even a drying period of 48 h did not lead to an *MC_res_* lower than 2% in almost every case. When an increased drying temperature of 80 °C was applied, the *MC_res_* could be reduced to less than 0.5%. Nevertheless, most of the specimens among those collectives being dried at 80 °C showed an *MC_res_* > 0.5%. Only drying at 103 °C for at least 24 h led to an *MC_res_* close to 0% (Table 1).

**Table 1:** Residual moisture content (*MC_res_* in %) after drying under defined conditions (temperature in °C, duration in h) in drying cabinets with air circulation. Mean *MC_res_* values plus corresponding standard deviation (in brackets) were summarized depending on the wood species and specimen format. Color change from dark green to dark yellow indicates increasing *MC_res_*, negative differences highlighted in blue. Intra-species-specific statistically significant differences related to the collective “103 °C, 48 h” are reported for the following levels: ^a^ *p* < 0.1; ^b^ *p* < 0.05; ^c^ *p* < 0.01.

Wood Species	Format (mm^3^)	60 °C				80 °C				103 °C			
		8 h	16 h	24 h	48 h	8 h	16 h	24 h	48 h	8 h	16 h	24 h	48 h
Norway spruce (*Picea abies*)	5×20×10	2.4 (1.1)	1.1 (0.4)	2.6 (0.2)	1.2 (0.2)	0.3 (0.5)	0.0 (0.7)	0.2 (0.9)	1.0 (0.4)	0.3 (0.4)	0.1 (0.4)	0.8 (0.4)	−0.7 (0.3)
	10×5×100	3.8 (0.4)	1.9 (0.3)	1.8 (0.1)	2.0 (0.1)	1.2 (0.2)	0.4 (0.1)	0.6 (0.0)	0.7 (0.1)	0.1 (0.0)	0.5 (0.1)	0.3 (0.1)	−0.3 (0.2)
	25×25×10	2.9 (0.2)	1.4 (0.1)	1.8 (0.1)	2.0 (0.1)	1.2 (0.2)	0.4 (0.1)	0.6 (0.0)	0.7 (0.3)	0.0 (0.2)	0.2 (0.1)	0.6 (0.1)	−0.6 (0.1)
	10×10×180	5.2 (0.3)	2.8 (0.2)	2.3 (0.2)	2.2 (0.1)	1.4 (0.2)	0.7 (0.1)	0.6 (0.1)	0.8 (0.1)	0.2 (0.2)	0.4 (0.1)	0.2 (0.0)	−0.3 (0.0)
	25×15×50	3.4 (0.1)	2.1 (0.0)	2.1 (0.2)	2.0 (0.0)	0.7 (0.1)	0.6 (0.0)	0.5 (0.0)	0.8 (0.2)	0.1 (0.1)	0.2 (0.1)	0.3 (0.1)	−0.4 (0.1)
	**MEAN**	**2.3 (1.0) ^c^**				**0.6 (0.5) ^c^**				**0.3 (0.3) ^c^**			**−0.5 (0.2)**
Scots pine SW (*Pinus sylvestris*)	5×20×10	2.1 (0.3)	2.3 (0.2)	2.2 (1.2)	1.8 (0.1)	-0.1 (0.2)	0.2 (0.3)	0.1 (0.8)	1.3 (0.2)	1.0 (0.3)	1.0 (0.7)	0.3 (0.2)	−1.0 (0.2)
	10×5×100	3.9 (0.6)	2.3 (0.2)	1.9 (0.1)	2.4 (0.2)	0.8 (0.3)	1.2 (0.9)	0.7 (0.1)	0.8 (0.1)	0.3 (0.0)	0.6 (0.1)	0.5 (0.2)	−0.5 (0.1)
	25×25×10	3.3 (0.2)	2.0 (0.2)	2.2 (0.9)	2.3 (1.2)	0.6 (0.2)	0.9 (0.2)	0.7 (0.2)	0.8 (0.4)	0.0 (0.1)	0.8 (0.1)	0.5 (0.1)	−0.6 (0.2)
	10×10×180	4.8 (0.3)	2.9 (0.1)	2.3 (0.1)	2.5 (0.2)	0.9 (0.1)	1.0 (0.1)	0.7 (0.0)	0.9 (0.1)	0.5 (0.0)	0.5 (0.1)	0.3 (0.0)	−0.4 (0.0)
	25×15×50	4.0 (0.2)	2.4 (0.1)	2.0 (0.0)	2.2 (0.1)	0.9 (0.1)	1.0 (0.0)	0.6 (0.0)	0.9 (0.0)	0.6 (0.1)	0.4 (0.0)	0.3 (0.1)	−0.4 (0.1)
	**MEAN**	**2.6 (0.9) ^c^**				**0.7 (0.4) ^c^**				**0.5 (0.3) ^b^**			**−0.6 (0.3)**
Scots pine HW (*Pinus* *sylvestris*)	5×20×10	4.2 (0.8)	3.5 (1.1)	2.1 (1.5)	3.1 (0.0)	2.2 (0.8)	3.2 (0.8)	1.3 (0.5)	1.3 (0.2)	1.2 (0.9)	1.5 (0.4)	0.4 (0.2)	−1.0 (0.3)
	10×5×100	3.9 (0.2)	2.6 (0.5)	2.9 (1.0)	2.6 (0.2)	4.0 (2.0)	2.1 (1.3)	0.7 (0.2)	1.0 (0.1)	1.1 (1.2)	0.8 (0.6)	0.2 (0.0)	−0.5 (0.2)
	25×25×10	3.7 (0.4)	2.4 (0.3)	1.9 (0.2)	2.7 (0.2)	1.3 (0.2)	1.4 (0.2)	0.8 (0.1)	1.4 (1.0)	0.4 (0.1)	0.7 (0.1)	0.5 (0.3)	−0.5 (0.2)
	10×10×180	5.6 (0.6)	4.2 (2.2)	2.7 (0.3)	3.5 (0.3)	2.1 (0.6)	1.4 (0.2)	0.7 (0.1)	1.1 (0.4)	0.8 (0.2)	0.5 (0.2)	0.3 (0.1)	−0.2 (0.1)
	25×15×50	4.3 (0.1)	2.5 (0.2)	2.0 (0.1)	2.6 (0.1)	1.2 (0.1)	1.3 (0.2)	0.8 (0.1)	0.9 (0.1)	0.5 (0.1)	0.4 (0.1)	0.3 (0.2)	−0.2 (0.1)
	**MEAN**	**3.2 (1.2) ^c^**				**1.5 (1.0) ^c^**				**0.6 (0.5) ^c^**			**−0.5 (0.3)**
European beech (*Fagus* *sylvatica*)	5×20×10	2.2 (0.1)	0.9 (0.1)	1.6 (0.3)	2.0 (0.1)	0.5 (0.1)	1.2 (0.7)	0.4 (0.5)	0.7 (0.2)	0.1 (0.2)	0.5 (0.3)	0.6 (0.3)	−0.3 (0.2)
	10×5×100	3.8 (0.2)	1.7 (0.1)	1.6 (0.1)	2.0 (0.0)	0.9 (0.1)	0.7 (0.1)	0.4 (0.0)	0.7 (0.0)	0.0 (0.0)	0.3 (0.1)	0.2 (0.1)	−0.2 (0.0)
	25×25×10	2.5 (0.1)	1.4 (0.1)	1.0 (0.2)	1.2 (1.3)	0.2 (0.0)	0.7 (0.1)	0.3 (0.2)	0.5 (0.1)	-0.2 (0.1)	0.5 (0.2)	0.3 (0.1)	−0.1 (0.1)
	10×10×180	4.9 (0.3)	2.7 (0.2)	2.0 (0.1)	2.0 (0.0)	1.5 (0.1)	0.7 (0.1)	0.5 (0.0)	0.6 (0.0)	0.2 (0.0)	0.4 (0.1)	0.2 (0.0)	−0.2 (0.0)
	25×15×50	4.5 (0.1)	2.2 (0.1)	1.7 (0.1)	2.0 (0.1)	1.0 (0.0)	0.7 (0.0)	0.4 (0.0)	0.5 (0.0)	0.2 (0.0)	0.1 (0.0)	0.2 (0.0)	−0.2 (0.0)
	**MEAN**	**2.2 (1.1) ^c^**				**0.7 (0.4) ^c^**				**0.2 (0.2) ^c^**			**−0.2 (0.1)**
Black locust (*Robinia pseudo-acacia*)	5×20×10	2.9 (0.5)	1.9 (0.2)	1.6 (0.3)	2.1 (0.1)	0.5 (0.2)	0.4 (0.2)	0.5 (0.1)	0.7 (0.1)	0.0 (0.2)	0.1 (0.2)	0.0 (0.2)	−0.3 (0.2)
	10×5×100	5.0 (0.2)	3.1 (0.1)	2.5 (0.2)	2.5 (0.0)	2.3 (0.1)	1.3 (0.2)	1.0 (0.1)	0.8 (0.1)	0.6 (0.1)	0.2 (0.1)	0.0 (0.0)	−0.1 (0.1)
	25×25×10	3.4 (0.2)	2.2 (0.1)	1.7 (0.0)	2.1 (0.0)	1.0 (0.1)	0.6 (0.0)	0.5 (0.0)	0.6 (0.1)	0.1 (0.1)	0.0 (0.0)	-0.1 (0.1)	−0.2 (0.0)
	10×10×180	6.2 (0.1)	4.7 (0.2)	3.7 (0.2)	3.2 (0.0)	3.7 (0.1)	2.5 (0.1)	1.7 (0.1)	2.4 (2.7)	2.1 (0.2)	1.0 (0.1)	0.4 (0.1)	0.1 (0.0)
	25x15x50	6.2 (0.2)	4.6 (0.2)	3.7 (0.3)	2.1 (1.1)	4.0 (0.3)	2.6 (0.2)	1.8 (0.1)	1.2 (0.1)	1.8 (0.2)	0.8 (0.1)	0.2 (0.1)	0.1 (0.0)
	**MEAN**	**3.3 (1.4) ^c^**				**1.5 (1.2) ^c^**				**0.5 (0.6) ^c^**			**−0.1 (0.2)**

### 3.2. Impact Factors on Density Measurements

Residual moisture contents (*MC_res_* in %) were determined for all combinations of drying temperature and duration compared with oven-drying at 103 °C for 48 h (3.1). It became evident that shortening the drying time as well as reducing drying temperatures led to incomplete drying, which as a consequence did immediately affect the oven-dry density calculated on the basis of the latter.

#### 3.2.1. Impact Factor Temperature and Duration

**Figure 2:**
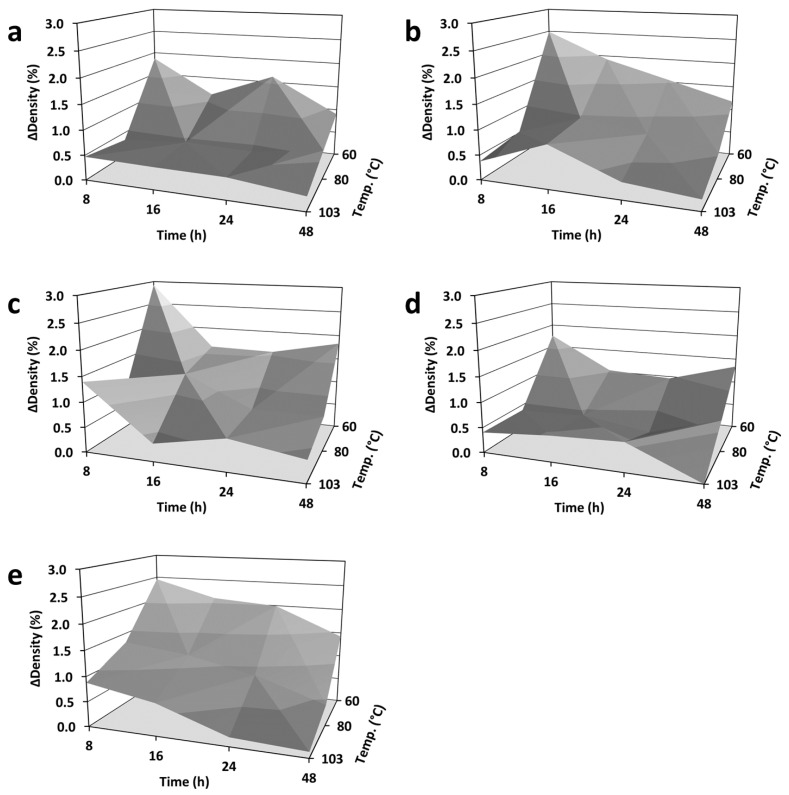
Interrelationship between density differences (*ΔDensity* in %) of 25 × 15 × 50 (ax.) mm^3^ specimens and drying conditions (temperature in °C, duration in h) applied in ventilated drying cabinets: (**a**) Norway spruce (*Picea abies*); (**b**) Scots pine sapwood (*Pinus sylvestris*); (**c**) Scots pine heartwood (*Pinus sylvestris*); (**d**) European beech (*Fagus sylvatica*); (**e**) Black locust (*Robinia pseudoacacia*).

The interrelationship between density differences (*ΔDensity* in %) and drying conditions are exemplarily shown in Figure 1 for the wood species of choice and the specimen format of 25 × 15 × 50 (ax.) mm^3^. In general, the accuracy of oven-dry density measurements increased with increasing drying temperature and duration, whereas the drying temperature appeared to have a stronger impact than the drying duration at a given temperature, which is shown in Figure 2. Drying wood specimens with a high content of volatile ingredients led to an apparently increased residual MC and density differences (e.g., Scots pine heartwood), as illustrated in Figure 2c. The latter can be attributed to the emissions of volatile organic compounds, which occur during drying processes at an elevated temperature (i.e., [51,52]). Such effects are expected to rise with increasing drying temperature [53,54], being even more pronounced in softwood than in hardwood species due to volatile terpene emissions as well as secondary emissions, such as of acetic acid and others [55]. Along most European softwood species, Scots pine (*Pinus sylvestris*) appears to be the softwood species which emits almost the highest concentrations of VOCs, predominantly monoterpenes (i.e., [56,57]). Thus, increasing amounts of volatile compounds have to be considered as an additional source of error for oven-dry density measurements on the basis of hot air drying processes at elevated temperature. Such error is expected to occur at its maximum for species with exceptionally high VOC concentrations, as has been shown for Scots pine in the present study.

#### 3.2.2. Impact Factor of Specimen’s Volume and Surface Area

**Figure 3:**
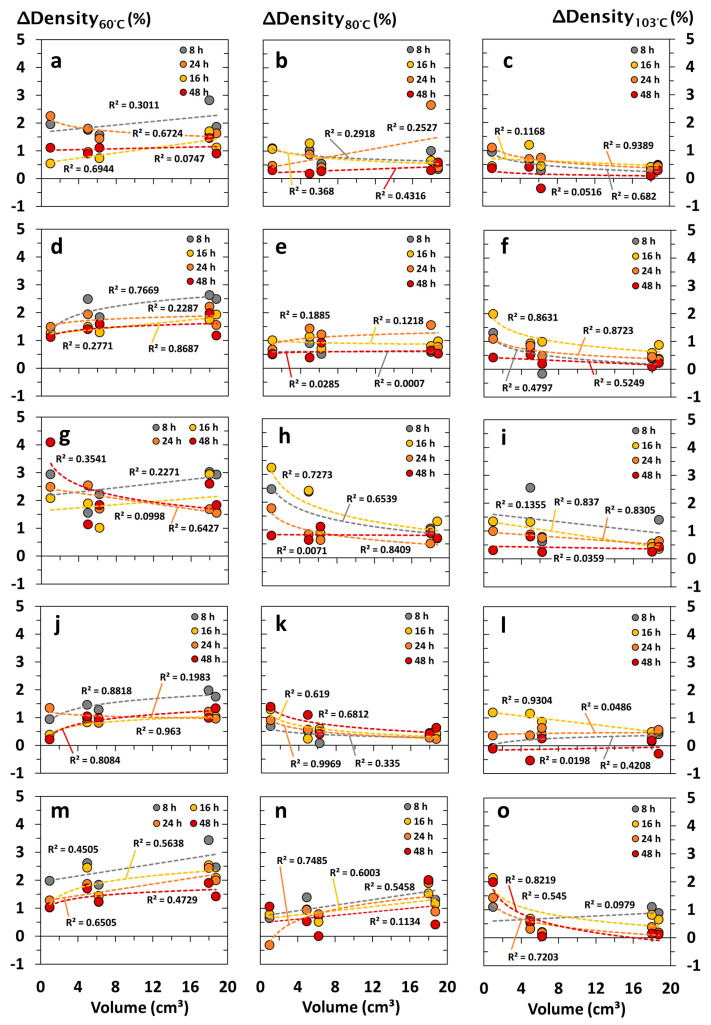
Interrelationship between density differences (*ΔDensity* in %) and the volume of wood specimens after drying under defined conditions (temperature in °C, duration in h) in drying cabinets: (**a**–**c**) Norway spruce (*Picea abies*); (**d**–**f**) Scots pine sapwood (*Pinus sylvestris*); (**g**–**i**) Scots pine heartwood (*Pinus sylvestris*); (**j**–**l**) European beech (*Fagus sylvatica*); (**m**–**o**) Black locust (*Robinia pseudoacacia*).

**Figure 4:**
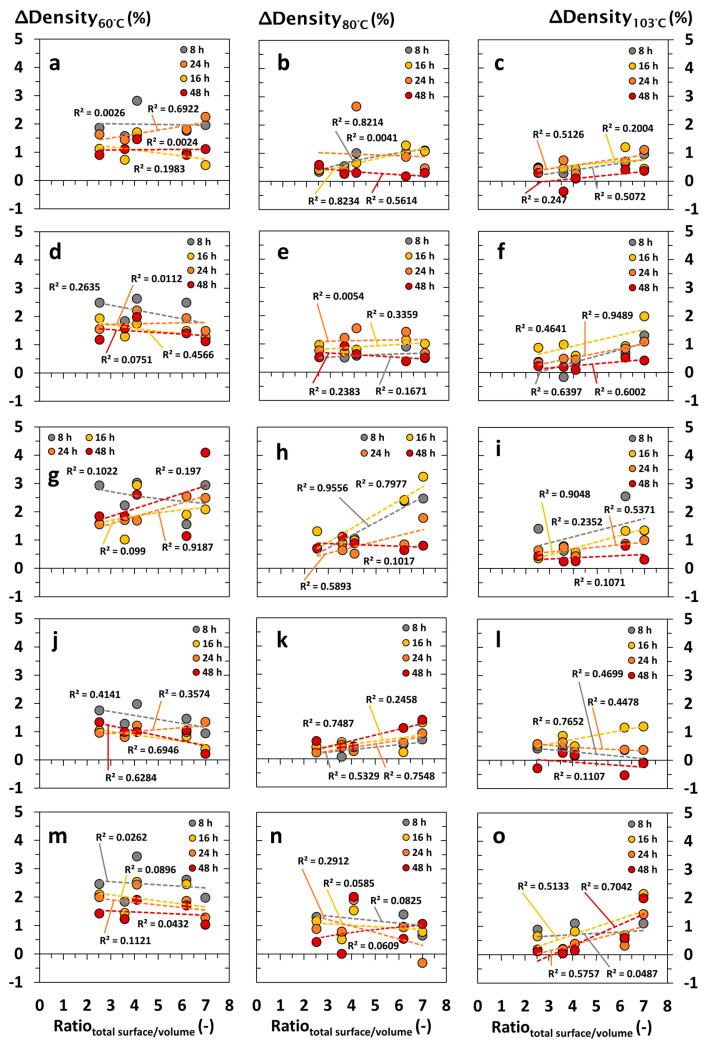
Interrelationship between density differences (*ΔDensity* in %) and the surface area–volume ratio of the wood specimens after drying under defined conditions (temperature in °C, duration in h) in drying cabinets: (**a**–**c**) Norway spruce (*Picea abies*); (**d**–**f**) Scots pine sapwood (*Pinus sylvestris*); (**g**–**i**) Scots pine heartwood (*Pinus sylvestris*); (**j**–**l**) European beech (*Fagus sylvatica*); (**m**–**o**) Black locust (*Robinia pseudoacacia*).

**Figure 5:**
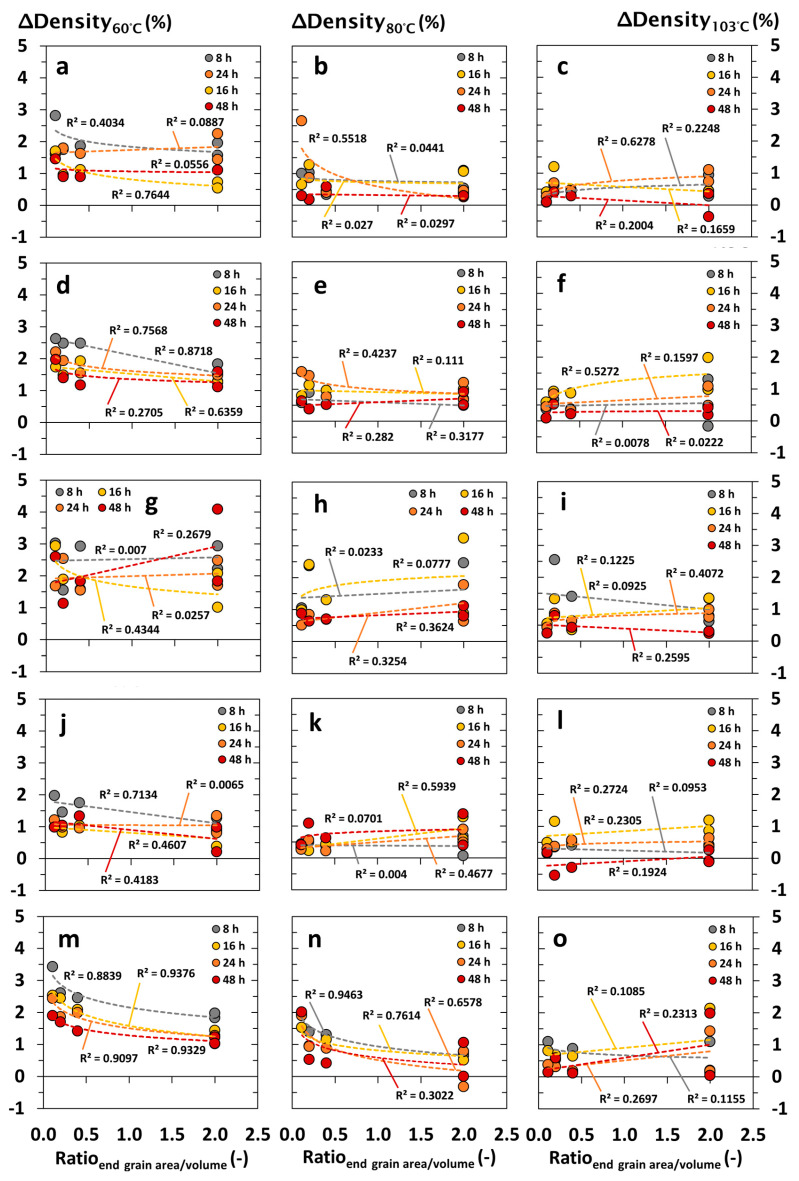
Interrelationship between density differences (*ΔDensity* in %) and the end grain surface area–volume ratio of the wood specimens after drying under defined conditions (temperature in °C, duration in h) in drying cabinets: (**a**–**c**) Norway spruce (*Picea abies*); (**d**–**f**) Scots pine sapwood (*Pinus sylvestris*); (**g**–**i**) Scots pine heartwood (*Pinus sylvestris*); (**j**–**l**) European beech (*Fagus sylvatica*); (**m**–**o**) Black locust (*Robinia pseudoacacia*).

**Figure 6:**
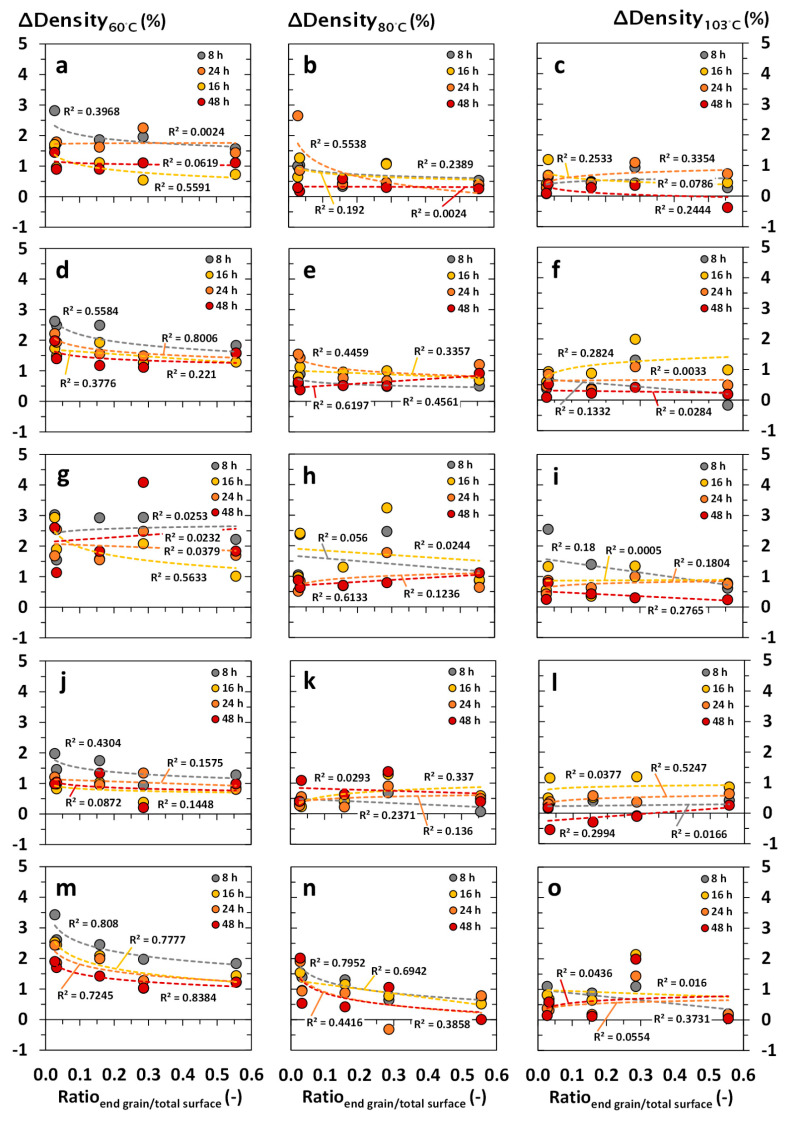
Interrelationship between the density differences (*ΔDensity* in %) and the end grain surface area–total surface area ratio of the wood specimens after drying under defined conditions (temperature in °C, duration in h) in drying cabinets: (**a**–**c**) Norway spruce (*Picea abies*); (**d**–**f**) Scots pine sapwood (*Pinus sylvestris*); (**g**–**i**) Scots pine heartwood (*Pinus sylvestris*); (**j**–**l**) European beech (*Fagus sylvatica*); (**m**–**o**) Black locust (*Robinia pseudoacacia*).

The interrelationship between the density differences (*ΔDensity* in %) and specimen formats of choice are summarized in Figure 3, Figure 4, Figure 5 and Figure 6 and illustrated depending on the drying temperature applied and wood species being tested. In principle, density differences (*ΔDensity* in %) decreased with increasing drying temperature and duration. In contrast, density measurements were negligibly affected by the specimen’s volume, showing no clear correlation with the *ΔDensity* (in %). The latter occurred independently of the wood species being tested, whereas wood species with a high content of volatile ingredients led to apparently increasing deviations of *ΔDensity* (in %), which were noticed at a decreasing drying temperature and specimen volume, as shown in Figure 3g–i. Independent of the drying temperature and duration, the total surface area of the specimens did not affect the accuracy of oven-dry density measurements at a significant level (Figure 4). Nevertheless, increasing end grain surfaces tended to decrease the density differences (*ΔDensity* in %), which were measured on the basis of dry mass and dimensions. The latter occurred even more pronounced at low drying temperatures of 60 and 80 °C, which is shown in Figure 5 and Figure 6 for both softwood and hardwood species. Principally, Scots pine heartwood, representing a wood species with a high content of volatile ingredients, showed significantly higher deviations in *ΔDensity* (in %) along the collective of tested wood species. Such effects increased with decreasing drying temperature and duration (Figure 3, Figure 4, Figure 5 and Figure 6).

## 4. Conclusions

The drying conditions (temperature in °C, duration in h) required to achieve the oven-dry state inside solid wood specimens that is obligatory for the analysis of various physical, mechanical, or even biological properties of wood-based materials were examined. The following was concluded from the obtained results:

- Oven-dry measurements (i.e., 48 h at 103 °C) themselves contain a significant error: up to 0.6% of the oven-dry weight in this study. The error is considered to be the result of deviations in the handling of the specimens and the scales used.
- Using temperatures below 103 °C and durations below 48 h led to significant deviations from the oven-dry weight obtained under reference conditions, i.e., 48 h at 103 °C. In particular, the use of 60 or 80 °C is considered critical for cases where absolute dry mass and dimensions should be measured. Therefore, drying at 103 °C occurred to be obligatory for an accurate determination of absolute dry mass and dimensions, consequently determining oven-dry density. The latter applies for the test specimens of choice and drying periods of ≤48 h.
- Drying wood specimens with a high content of volatile ingredients led to apparently increased residual MC values (e.g., shown for Scots pine heartwood), which led to inaccurate oven-dry density calculations. Thus, volatile ingredients were considered an additional source of error during oven-dry measurements and required a differentiated analysis of further drying conditions under the consideration of changes in the chemical composition and structure of the structural cell wall components.

## Data Availability

The original contributions presented in this study are included in the article. Further inquiries can be directed to the corresponding author.
